# Discussing the gaps in the science and practice of lived experience engagement in mental health and substance use research: results of knowledge mobilization activities

**DOI:** 10.1186/s40900-024-00554-6

**Published:** 2024-02-10

**Authors:** Lisa D. Hawke, Faith Rockburne, Melissa Hiebert, Connie Putterman, Natasha Y. Sheikhan

**Affiliations:** 1 Centre for Addiction and Mental Health, 60 White Squirrel Way, Toronto, ON Canada; 2 Department of Psychiatry, University of Toronto, 60 White Squirrel Way, 316, Toronto, ON M6J 1H4 Canada

**Keywords:** Lived experience, Patient-oriented research, Research methods

## Abstract

**Background:**

Engaging people with lived experience of mental health or substance use challenges and family members (PWLE) improves the quality and relevance of the associated research, but it can be challenging to include them meaningfully and authentically in the work.

**Knowledge mobilization events:**

After reviewing the literature on the science of lived experience engagement, we held two knowledge mobilization events to translate the findings to relevant partners and collect their feedback to guide our future research. A total of 55 people attended, bringing the perspective of people with lived experience, family members, research staff, research trainees, and scientists, as well as attendees holding multiple roles. We presented the scoping review findings, then held discussions to solicit feedback and encourage the sharing of perspectives.

**Attendee perspectives:**

Through small and large group discussion activities, we found that our scoping review findings resonated with the attendees’ personal experiences with engagement in mental health and substance use research. Among the gaps highlighted in the discussions, the two that were most emphasized were the critical importance of improving diversity in engagement work in mental health and substance use, and the importance of addressing gaps around communication, relationships, rapport, and power dynamics in engagement spaces.

**Conclusions:**

Diversity, communication, relationships, and power dynamics emerge as key areas of work needed in the near future to advance the science of PWLE engagement in mental health and substance use research. We commit to pursuing the work that is considered of greatest need by a range of partners this research engagement sphere. We call on researchers in this area to continue this line of work, with a focus on the areas of research identified by attendees.

## Abstract (plain language summary)

Engaging people with lived experience of mental health or substance use challenges and family members (PWLE) improves research, but it can be challenging to do it right. We reviewed the scientific research on engaging people with lived experience in this area of research. Next, we held two community events to share our findings and brainstorm on next steps. A total of 55 people attended. They were people with lived experience, family members, research staff, research trainees, and scientists, as well as people coming from multiple perspectives. Through small and large group discussions, attendees talked about the findings and the next steps. As a whole, attendees recognized the importance of this area of work and largely agreed with the findings we presented. Among all of the material discussed, attendees emphasized 1) the importance of improving diversity in PWLE engagement, and 2) the importance of improving communication, relationships, rapport, and power dynamics. These two key issues are considered among the most important areas of work going forward to advance the science of PWLE engagement in mental health and substance use research. We commit to pursuing the work that is considered of greatest need and call on researchers in this area to continue this line of work, with a focus on the areas of research identified by attendees.

## Background

Engaging people with lived experience (PWLE) of mental health or substance use challenges, including family members, improves research quality and relevance, but can be challenging to do meaningfully and authentically [1]. As part of our work to advance the science of PWLE engagement in mental health and substance use research, we conducted a scoping review of the literature to understand research evidence and implementation gaps, published in *Research Involvement and Engagement* [2]. That review identified a range of research gaps: conceptualizing engagement, developing engagement resources, increasing diversity, and evaluating engagement. Implementation gaps included broader institutional gaps such as funding concerns, ethics board issues, and institutional supports, as well as concrete day-to-day practice gaps such as providing training and mentorship, building relationships and rapport, increasing diversity in practice, planning, and embedding engagement in leadership. Gaps reflect many of the barriers and facilitators to engagement, as identified across the mental health and psychiatry research literature, where similar institutional and practice gaps consistently emerge [1, 3–5].

## Knowledge mobilization events

Based on our scoping review, we held two knowledge mobilization events to translate the findings to end users and collect their feedback. The project encompassed deliberative and consultative engagement [6] (See Table 1 for the GRIPP-2 checklist [7]). The project was approved by the Quality Project Ethics Review (#QPER-39) team at the Centre for Addiction and Mental Health (CAMH).

**Table 1:** Guidance for reporting involvement of patients and the public (GRIPP2) reporting checklist for lived experience engagement in research [7]

Section and topic	Description
1: Aim	This knowledge mobilization project aimed to garner the feedback of people with lived experience (PWLE), including family members, by holding events in which PWLE were engaged at all stages.
2: Methods	Project planning and funding acquisition occurred with a PWLE advisory group, with two PWLE as co-applicants for funding. One PWLE co-facilitated the events. A patient engagement in research coordinator with personal lived experience contributed to the development and facilitation of the event. A family engagement in research coordinator, with expertise in research engagement and family partner experience, further contributed to event development and facilitation. The results were brought back to the PWLE advisory group for discussion and brainstorming about next steps. One person in a PWLE role is co-author of this report, alongside team members with multiple perspectives, including research and lived experience roles.
3: Study results	The project plan was developed in a manner that resonated with PWLE. The event recruitment and presentation materials were co-developed with PWLE, for clear objectives and presentations. An engaging facilitation style was utilized, through PWLE co-facilitation. PWLE feedback acquired during the events was synthesized together with feedback from people bringing other perspectives. The post-event reflections of PWLE were integrated into the next steps for this line of work.
4: Discussion and conclusions	PWLE engagement shaped all aspects of the project, from the development of the initial idea and funding acquisition process to the final reporting. This encompassed deliberative and consultative engagement. The results and interpretations represent the perspectives of PWLE, together with those of other relevant partners with whom they work in engagement contexts.
5: Reflections/critical perspective	This project was guided by lived experience at all levels of planning, execution, and completion, which was pivotal to the project’s success.

The two knowledge mobilization events, held in fall 2023, brought together 55 attendees including PWLE, research staff, family members, lead scientists, research trainees, and individuals with multiple perspectives. The events were developed with the support of a Lived Experience Advisory Group and were publicized through the team’s contacts, knowledge user newsletters, and social media posts. The first event (22 attendees) was held in person at CAMH. The second event (33 attendees) was conducted virtually. After an introduction and a brief presentation of the scoping review, we held facilitated discussions using the World Café method [8], which combines large group discussions with smaller break-out groups. Facilitators were a scientist, a PWLE, and patient and family engagement coordinators. Discussions focused on attendees’ perspectives on our findings and their thoughts on the aspects of PWLE engagement most urgently requiring attention. After the events, the feedback was narratively synthesized from participant and facilitator notes. Findings were reported back to a Lived Experience Advisory Group for feedback and discussion.

### Findings

Across events, attendees recognized the importance of advancing the science of PWLE engagement. Attendees were pleased that this work was being conducted. They expressed that the scoping review findings resonated with them, reflecting challenges and gaps that they have encountered.

Among the evidence and implementation gaps presented, some of the most notable concerns of attendees centered on the importance of enhancing diversity in PWLE engagement spaces. A key topic that arose from the discussion included increasing diversity across a wide range of sociodemographic and mental health variables, including various communities and individuals with different characteristics across racial/cultural background, age, gender, and diagnosis or mental health/substance use challenge, with attention to intersectionalities among them [9]. Diversity should be increased through active outreach, by increasing awareness of opportunities among people with lived experience and family members, and by ensuring that engagement activities are inclusive, accessible and trauma-informed in order to engage diverse and vulnerable people. Another dominant area of discourse was the need to work on communications, relationships, rapport, and power. Participants highlighted the critical importance of explaining research concepts clearly, actively listening and authentically valuing the perspectives of PWLE, compensating PWLE for their work, and fostering opportunities for consistent, ongoing engagement. Other factors were highlighted, with less emphasis. These included funding issues, planning, recruitment, budgeting, describing best practices, describing both good and bad engagement experiences, embedding engagement throughout institutions and in leadership positions, and conducting rigorous research and evaluation of the engagement process and outcomes for various key individuals, including PWLE. These areas of emphasis by attendees inform us about the most important directions for the science of PWLE engagement in mental health and substance use research moving forward.

In a post-event Lived Experience Advisory Group meeting, members reflected on the events and brainstormed on next steps. The importance of pursuing funding for projects to better understand diversity in PWLE engagement, across a wide variety of characteristics, was highlighted. Possible directions included understanding the current profiles across multiple sociodemographic characteristics including intersectionalities and querying diverse engaged PWLE about means of facilitating entry into engagement spaces.

### Evaluation

The events were evaluated using the Patient and Public Engagement Evaluation Tool (PPEET) [10]. The PPEET is evaluated on a 1–5 Likert scale, where 1 represents ‘strongly disagree,’ and 5 represents ‘strongly agree.’ Thirty-five attendees completed the PPEET, including 14 PWLE, 4 family members, 9 from research or research trainee perspectives, and 8 attendees bringing multiple perspectives (e.g., research and lived experience) or holding other roles. The average score was 4.2 (SD = 0.57), i.e., above ‘Agree’ on the Likert scale, with a range of 2.5 to 5.0. Subscale scores were M = 4.0 (SD = 0.80) for *Communication and Supports for Participation*, 4.4 (SD = 0.61) for *Sharing your Views and Perspectives*, 4.1 (SD = 0.60) for *Impacts and Influence of the Engagement Initiative*, and 4.2 (SD = 0.76) for *Final Thoughts*. These results suggest that participants generally felt positive about the events.

### Strengths and limitations

This work was conducted with PWLE engagement at project leadership, the advisory level, and in the dissemination events, providing a range of engagement from consultation through to leadership [6]. While it was a relatively small project, we brought together people in various relevant roles for joint listening, discussion, and brainstorming. Some PWLE attendees were interested in sharing their perspectives on clinical concerns, which could not be fully accommodated within the agenda. Since this was not research, complete demographic information is not available and it is unclear to what extent the attendees had experience with authentic engagement. It is likely that the knowledge dissemination component of the event influenced the opinions of the individuals consulted, which may have limited the generation of novel ideas and could therefore constitute a bias. While the use of small breakout rooms enhanced attendees’ ability to have their voice heard, it is possible that group effects limited the breadth of the discussion. Self-selection for attendance may have constituted an additional bias, as individuals more engaged in this type of work may have been more likely to attend. Future projects might consider evaluating the difference between in-person and virtual versions of this type of event in terms of communication and findings. Nevertheless, the evaluation was positive, suggesting that the events were a successful means of sharing information with the target group and gaining their feedback.

## Conclusions

Through two knowledge mobilization events, we solicited the views of relevant partners in PWLE engagement in mental health and substance use research regarding the work that needs to be conducted to advance the science of PWLE engagement. Diversity, communication, relationships, rapport, and power dynamics emerged as a strong emphasis. We commit to pursuing the work that is considered of greatest need. We further call on researchers in this area to continue this line of work, with a focus on the areas of research identified by attendees.

## Data Availability

The post-event evaluation dataset reported on is available from the corresponding author on reasonable request, pending institutional approval. No other datasets were generated in the course of this work.

## Abbreviations

CAMH: Centre for addiction and mental health
PPEET: Patient and public engagement evaluation tool
PWLE: People with lived experience and family members
